# Racism and mental health in higher education: A challenge for LMICs

**DOI:** 10.1002/mpr.1799

**Published:** 2019-12-29

**Authors:** Vagner Dos Santos, Sara Leon Spesny, Sharon Kleintjes, Roshan Galvaan

**Affiliations:** ^1^ Ceilândia Faculty University of Brasília Brasília Brazil; ^2^ Institut de Recherche Interdisciplinaire sur Les Enjeux Sociaux École des Hautes Études en Sciences Sociales Paris France; ^3^ Division of Intellectual Disability, Department of Psychiatry and Mental Health University of Cape Town Cape Town South Africa; ^4^ Division of Occupational Therapy, Department of Health and Rehabilitation Sciences, Faculty of Health Sciences University of Cape Town Cape Town South Africa

As the authors of the article “Viewpoint: WHO World Mental Health Surveys International College Student Initiative: Implementation issues in low‐ and middle‐income countries” (LMICs) have pointed out, the university system and the number of students are rapidly growing and changing in LMICs (Evans‐Lacko & Thornicroft, 2019). In section 3, “Mental Health Consequences of Universities in Transition in LMICs,” the authors mention important elements of such social change that has led to a new generation of students in college and their challenges, including the lack of financial and family support to pursue their higher education. The authors identify important contextual factors which require consideration prior to the adaptation of interventions for university students, for example, ethical and cultural issues. In our view, it would be valuable to consider the influence of issues related to social identity—for example, LGBTIQ+, race, and disability—on the mental health of university students. Our letter intends to introduce another strand to the dialogue on issues in LMICs, specifically highlighting a commonly neglected aspect of the institutional life that also influences students' mental health within the higher education systems in LMICs—Racism.

Although racism is known to have a causal link with morbidity, disability, and mortality, this issue is not commonly addressed in public health or mental health agendas (Bailey et al., 2017; King, 2019). Racism affects students' well‐being, access, and performance in higher education. For instance, in Brazil, the country's myth of racial democracy has been widely criticized, and race has become a fundamental dimension in determining people's life chances and opportunities (Souza, 2017, 2018), including their experience of higher education (Bernardino‐Costa & Blackman, 2017; Hogan, de Araujo, Caldwell, Gonzalez‐Nahm, & Black, 2018). Brazil was the last country to abolish black slavery (in 1888) and has a large black population that faces several social injustices, including limited access to higher education and racist dynamics within campuses. Very recently, the number of black students has increased in Brazil due to the implementation of quotas and affirmative action policies in the higher education system throughout the country (Schwartzman & Paiva, 2016). Nonetheless, these affirmative action schemes have also been a source of debate and are subject to questioning from conservative sectors.

Likewise, South Africa has an ongoing challenge to reconcile a society strongly harmed by racism. At the end of the apartheid system, the early 1990s started a process of liberation; however, in what is known as the Soudien Report, a Ministerial Committee on Transformation and Social Cohesion and the Elimination of Discrimination in Public Higher Education still found discrimination based on gender and race to be endemic in public higher education institutions (Soudien et al., 2008). More recently, evidence of this systemic discrimination was brought into sharp relief through protests by groups of students and academics during the calls for free, decolonized higher education during 2015 and 2016. The Institutional Reconciliation and Transformation Commission (IRTC) at the University of Cape Town (UCT) was tasked with investigating allegations of racism and violence during the student protests and making recommendations to address unjust discrimination within the university. In March 2019, the IRTC released its final report (IRTC, 2019; Nordling, 2019), which acknowledges and describes racist dynamics at UCT and how these dynamics affect students' mental health. Interestingly, the Committee also points out that racism affect not only students' access and performance in higher education but also their mental health; they recommend that the university address this issue by providing appropriate mental health care.

We support Evans‐Lacko and Thornicraft's (2019) contention that responses need to go beyond service provision to address contextual factors in a full response to students' mental health needs. However, we add that this contextual response should include considerations of institutional racism. The contextually situated nature of psychological distress emphasizes the need for a human paradigm that recognizes how social determinants contribute to well‐being (Bantjes, Swartz, & Cembi, 2018). Narratives of the experiences of black female academics (Khunou, Phaswana, Khoza‐Shangase, & Canham, 2019) and university students (Swartz et al., 2018) in South Africa reveal how institutional discrimination based on race and gender has burdened those most affected. Evans‐Lako and Thornicraft (2019) emphasize the importance of one's socioeconomic position to mental health status, and in post‐colonial Brazil and post‐apartheid South Africa, it has to be acknowledged that socioeconomic positionality is inextricably linked to race (Pereira, 2016; South African Government, 2018).

Higher education systems in Brazil and South Africa leave black students and academics with experiences of alienation, limiting a healthy experience of belonging for black students within their campuses. Nonetheless, racist experiences among students have also been acknowledged as an issue in other large LMICs (Laruelle, 2010; Rahim, 1998); thus, further analysis of how racism affect students' mental health is needed. Finally, as academics, we have a fundamental role in acknowledging that the experiences of these students need to be better understood, including the suffering related to institutional racism in higher education, especially in LMICs.
